# Differential synapse density between Purkinje cell dendritic spine and parallel fiber varicosity in the rat cerebellum among the phylogenic lobules

**DOI:** 10.1186/s42649-020-00027-6

**Published:** 2020-02-27

**Authors:** Hyun-Wook Kim, Seung Hak Oh, Se Jeong Lee, Ji eun Na, Im Joo Rhyu

**Affiliations:** 1 Department of Anatomy, Korea University College of Medicine, Seoul, 02841 Korea; 2 Division of Brain Korea 21 Plus Program for Biomedical Science, Korea University College of Medicine, Seoul, 02841 Korea

**Keywords:** Cerebellum, Synapse, Phylogeny, Evolution

## Abstract

The cerebellum is a region of the brain that plays an important role in motor control. It is classified phylogenetically into archicerebellum, paleocerebellum and neocerebellum. The Purkinje cells are lined in a row called Purkinje cell layer and it has a unique dendritic branches with many spines.

The previous study reported that there is a difference of synapse density according to the lobules based on large two-dimensional data. However, recent study with high voltage electron microscopy showed there was no differences in dendritic spine density of the Purkinje cell according to its phylogenetic lobule. We analyzed Purkinje cell density in the II, VI and X lobules by stereological modules and synaptic density was estimated by double disector based on Purkinje cell density in the molecular layer of each lobule.

The results showed that there was significant difference in the Purkinje cell density and synapse number according to their phylogenetic lobules. The number of Purkinje cell in a given volume was larger in the archicerebellum, but synapse density was higher in the neocerebellum.

These data suggest that cellular and synaptic organization of the Purkinje cell is different according to their phylogenetic background.

## Introduction

The cerebellum is a key structure for controlling movement with integration of sensory and motor information. Specifically there are many reports on cerebellar function in motor control, learning and memory, sensory information processing (Gilbert and Thach 1977).

The cerebellum could be divided into archicerebellum, palecerebellum and neocerebellum by functional phylogenetic differentiation. The anatomical location of the anterior lobe is called the palecerebellum, the posterior lobe is called the neocerebellum and the flocculonodular lobe is called the archicerebellum. The archicerebellum is associated with the vestibular system, which controls the sense of balance. The palecerebellum is responsible for the gait function of harmonious movement and develops from more than four-legged reptiles. Neocerebellum is a relatively delicate motor control region that develops mainly in vertebrates (Larsell 1952).

Recently, lobule-specific membrane excitability of cerebellar Purkinje cell was reported based on electrophysiological study (Kim et al. 2012). The firing pattern and membrane properties of Purkinje cell in cerebellar lobule III-V was different from cerebellar lobule X. Furthermore, mossy fiber input were different in lobule VI/VII than lobule X, which finally propagate to signals to Purkinje cells accordingly (Witter and De Zeeuw 2015). This functional difference in electrophysiological research reminds the study on synapse difference in Purkinje cell according to their lobules. The synaptic density difference of Purkinje cell according to their phylogeny was reported based on transmission electron microscopic analysis (Heinsen and Heinsen 1983a, 1983b). But, there was not difference in dendritic spine density in Purkinje cell according to the cerebellar lobule (Park 2007), which was not in agreement with previous study.

In this study, we used stereology to understand phylogenetic differences in cerebellum exactly. The number and volume of Purkinje cells and the number of synapses between dendritic spine and parallel fiber are measured using optical microscope with electron microscope.

## Materials and methods

### Sampling

**Fig. 1:**
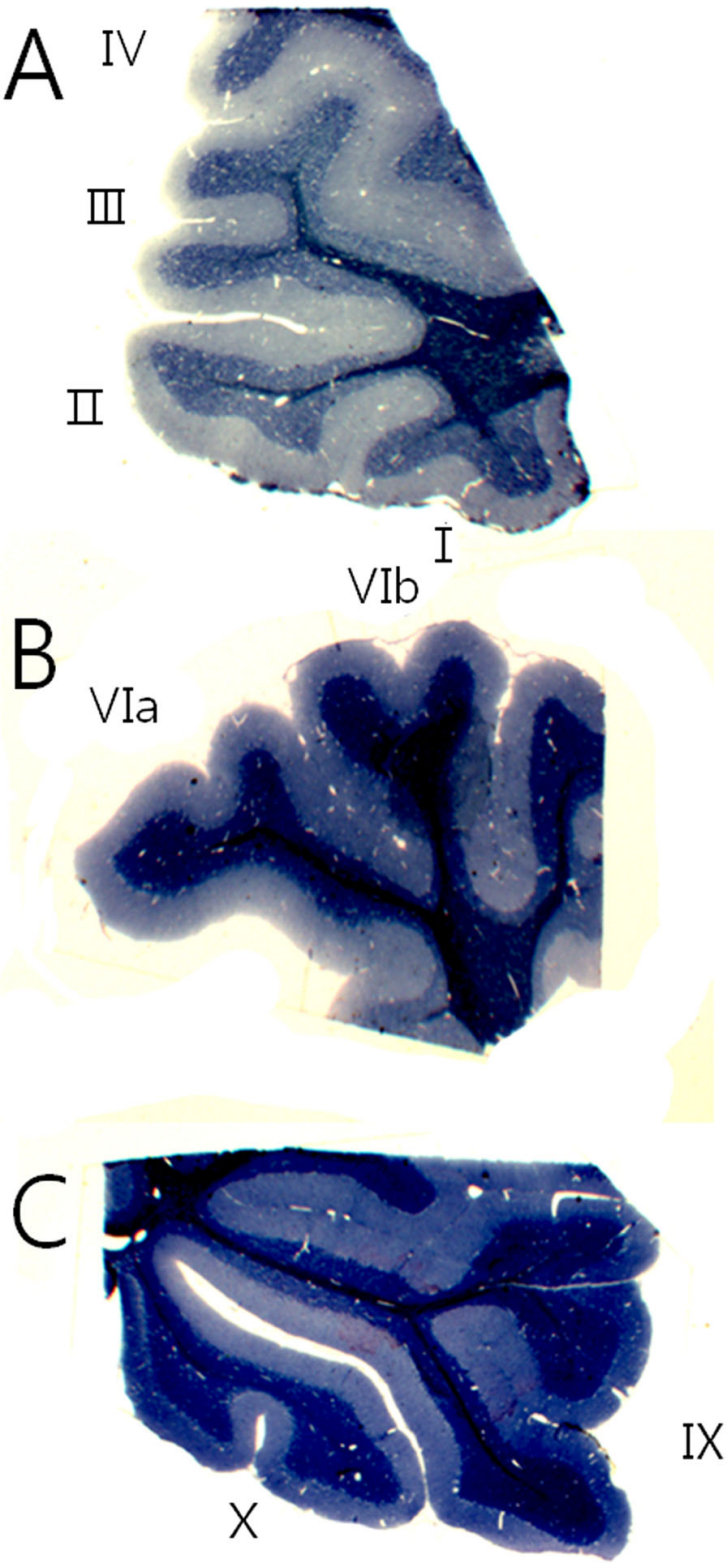
Image of sagittal section of the cerebellar vermis. The cerebellum is classified phylogenetically into archicerebellum, paledcerebellum and neocerebellum. The images based on light microscopy according to lobule. X100

Six-week-old Sprague Dawley male rats (*n* = 5) were anesthetized intraperitoneally with pentobarbital (100 mg / kg), followed by cardiac perfusion with fixative (4% paraformaldehyde with 1% glutaraldehyde 0.1 M phosphate buffer, pH 7.4). The extracted cerebellum was fixed for 24 h at same fixative at 4 °C and washed with 0.1 M phosphate buffer saline (PBS, pH 7.4). After then, the vibratome was used to obtain cerebellar serial sections of 150 μm along the sagittal-plane. Serial sections were trimmed by dividing into the palecerebellum (II), neocerebellum (VIb), and archicerebellum (X) (Fig. 1). Using the EM Tissue processor (Leica, Germany), post fixation was performed for 90 min with 1% osmium tetroxide and en bloc staining with 2% uranyl acetate. The tissues were dehydrated stepwise in increasing concentration of ethanol and substitution with propylene oxide. Substituted samples were embedded in EPON 812 epoxy resin and polymerized for 48 h in a 60 °C dry oven.

### Stereological analysis

#### Volume measurement of each cerebellar lobule in vermis

**Fig. 2:**
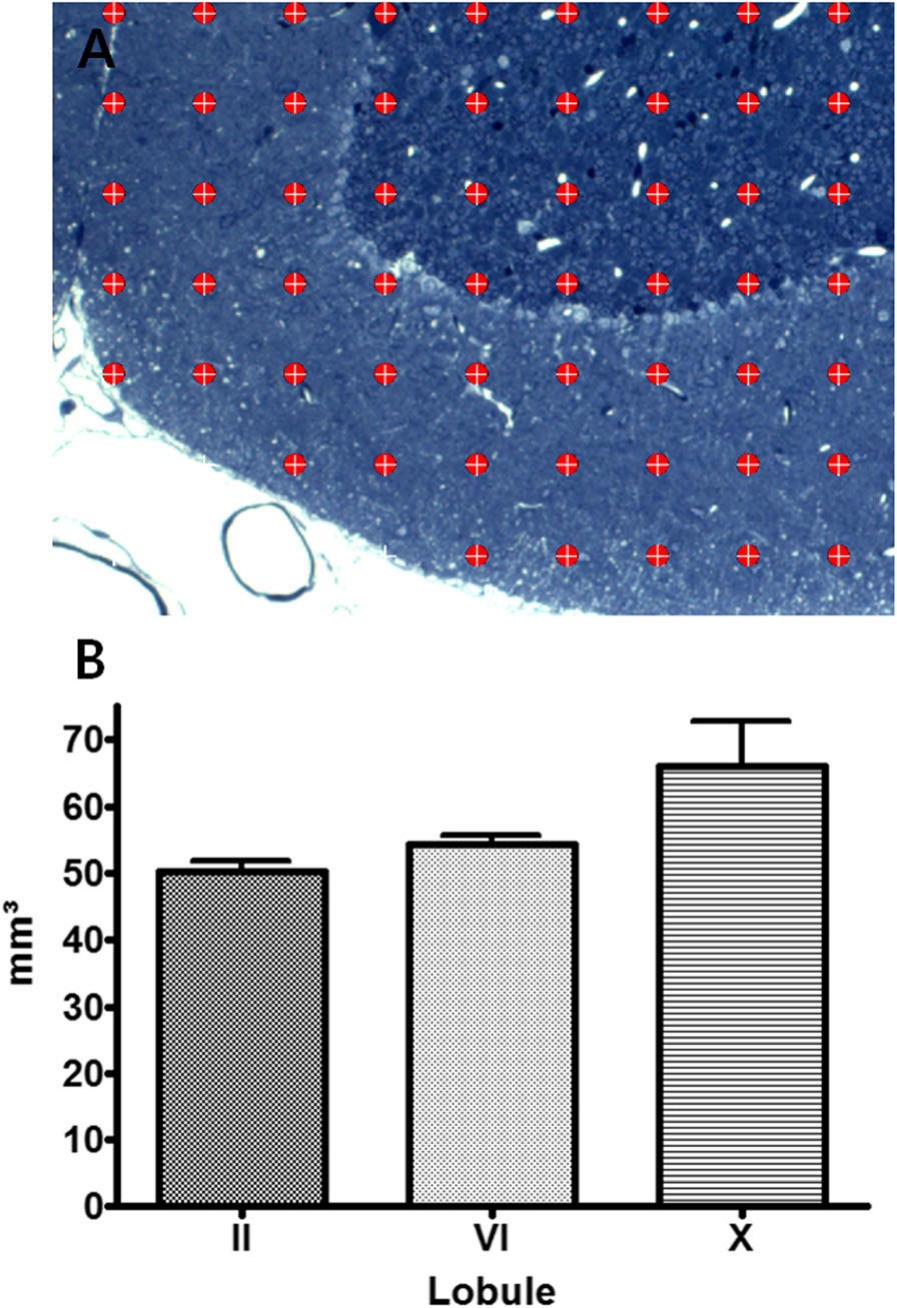
Cavalieri’s estimation of volume in cerebellum vermis. **a** Image is checked by overlaying a rectangular lattice of point on the area of each lobule. Grid scale is 50 μm, × 100, **b** Result from the Cavalieri probe of stereological analysis

According to the phylogenetic lobule, serial semi-thin sections (1 μm thickness) were obtained and stained with toluidine blue. Stained samples were observed under an optical microscope (Microimaging GMBH 37081, Carl Zeiss, Germany) and measured by a Cavalieri’s estimator method using a stereo investigator (Ver. 7.1, MBF, USA) (Michel and Cruz-Orive 1988). Cavalieri’s estimator is a method of calculating the three-dimensional volume of a structure through a serial section image of a tissue to be measured. The calculation is to multiply the sum of the areas (∑ A) by the thickness (T; 150 μm) in a serial section with each cerebellar lobule. The area was measured by using a grid of regular intervals (502 μm) on the image, measuring the intersections of overlapping molecular and granule layers of the lobule and can be calculated by the following formula (Fig. 2a).

$$ \mathrm{V}=\sum \mathrm{A}\ \mathrm{x}\ \mathrm{T} $$

#### Measurement of Purkinje cell density

To measure the number of Purkinje cells per unit area, two randomly selected blocks in each lobules, and then 40 serial sections of 1 μm thickness were collected slide glass, stained with toluidine blue, and obtained using an optical microscope. Sections were selected randomly and then unbiased random quantitative analysis was performed by physical disector method (Klintsova et al. 2002). Images are aligned by the Z axis using 3D Reconstruct software (http://synapses.clm.utexas.edu / tools), and then randomly arranged by setting a counting frame (300 × 150 μm), The cells on the forbidden line were not measured, only the cells on the acceptance line were measured. In addition, if visible in the reference section but not in the look up section, it means that the Purkinje cell is finished between the spaces of the two sections.

The number of cells measured using this method was calculated by the following formula.

$$ N=\frac{\mathrm{total}\ \mathrm{number}\ \mathrm{of}\ \mathrm{counted}\ \mathrm{cell}\ }{\mathrm{sum}\ \mathrm{of}\ \mathrm{area}\ \mathrm{of}\ \mathrm{disector}\ \mathrm{frame}} \times section\ thickness $$

#### Measurement method of synaptic density

**Fig. 3:**
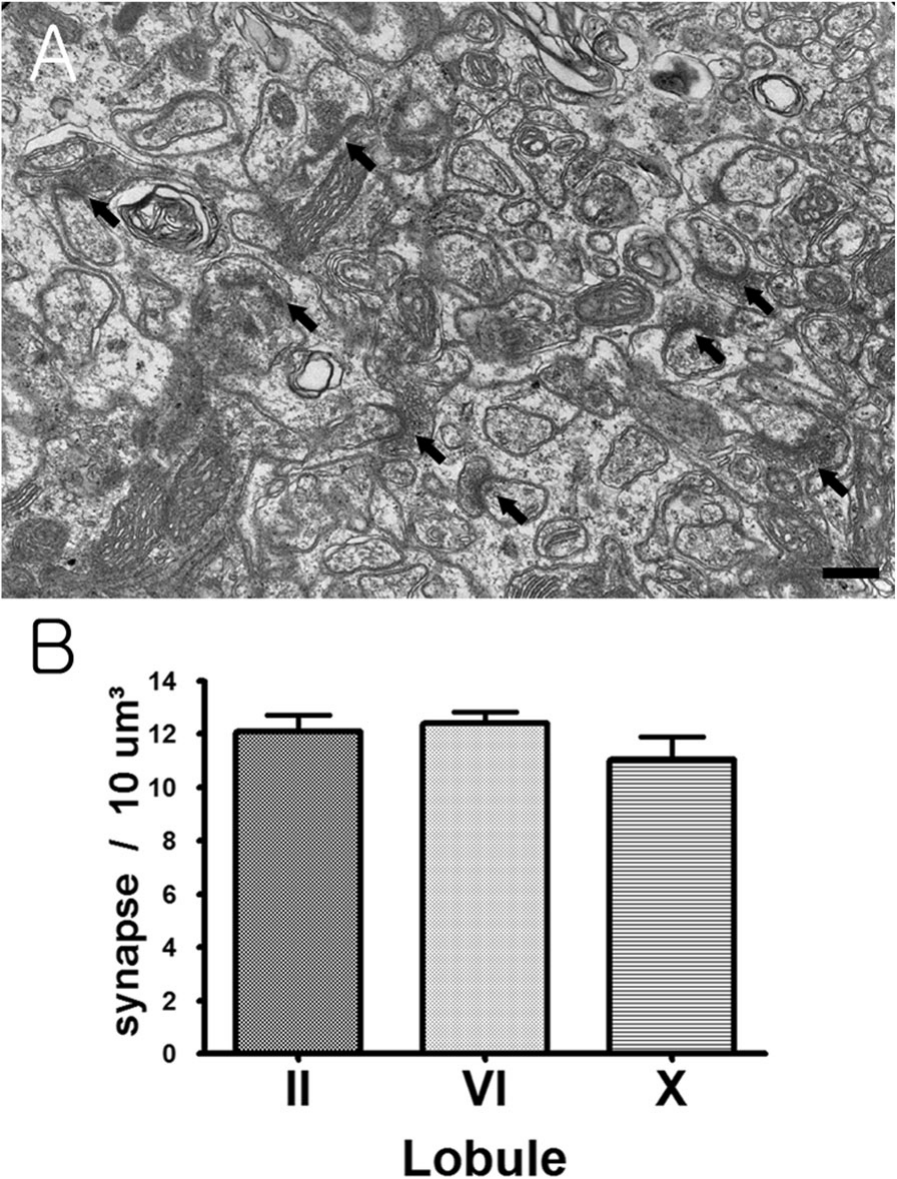
Transmission electron microscopy image of synapse and estimation of synapse density. This shows Purkinje cell dendritic spine synapse of molecular layer in cerebellum. Used transmission electron microscopy. **a** Indicate is synapse of molecular layer in cerebellum (arrows). × 60,000 Bar = 1 μm. **b** Histogram of synaptic density in the molecular layer

Among the molecular layers of the cerebellar vermis, a middle layer with many second branches of the Purkinje cell dendrite was selected to obtain a serial section of 80 nm thickness for synapse analysis. The serial section was double-stained with uranyl acetate and lead citrate on a single-slot grid with formvar membrane and carbon coating. Grids were observed at 15,000 magnification on a transmission electron microscope (H-7500, Hitachi, Japan). The results obtained were aligned along the Z axis in the 3D Reconstruct software. Using a constant frame (5.7um × 2.8um), the number of synapses was measured in the same method (Fig. 3a), and was calculated by the following formula.

$$ \mathrm{S}=\frac{\ \mathrm{total}\ \mathrm{number}\ \mathrm{of}\ \mathrm{counted}\ \mathrm{synased}\ }{\mathrm{sum}\ \mathrm{of}\ \mathrm{area}\ \mathrm{of}\ \mathrm{disector}\ \mathrm{frame}} \times \mathrm{section}\ \mathrm{thikness} $$

#### Estimation of synaptic density in single Purkinje cell

In order to measure the synapse number of single Purkinje cell in II, VIb, and X lobule, the number density of synapses per unit area measured previously was divided by the number density of Purkinje cells (Gundersen et al. 1988; Klintsova et al. 2002).

$$ \mathrm{N}\ \mathrm{synapses}\ \mathrm{per}\ \mathrm{PC}=\frac{\ \mathrm{Nv}\ \left(\mathrm{syn}\right)\ }{\mathrm{Nv}\ \left(\mathrm{PC}\right)\ } $$

### Statistical analysis

The volume density was calculated using a stereo investigator, and the number density was analyzed by Microsoft Excel (Microsoft, USA) based on the calculated value. All data were validated by repeated-measure ANOVA with *P* < 0.05 as the difference between leaflets (SPSS, IBM, USA).

## Result

### Volume of each lobule in cerebellar vermis

One hundred fifty micrometer thick samples of II, VIb, and X lobules were obtained with semi-thin sections of 1 μm each, and grids of 50 μm intervals were set in 17 serial sections per lobule. At this time, measure the number of grids overlapping with the lobule of Purkinje cell, multiply by the sum of the area (∑ A). The total area was the largest in the X lobule. However, there was no significant difference between the volume density of each lobule by One way ANOVA (*P* < 0.141) (Fig. 2b).

### Number density of Purkinje cell in each lobule

**Fig. 4:**
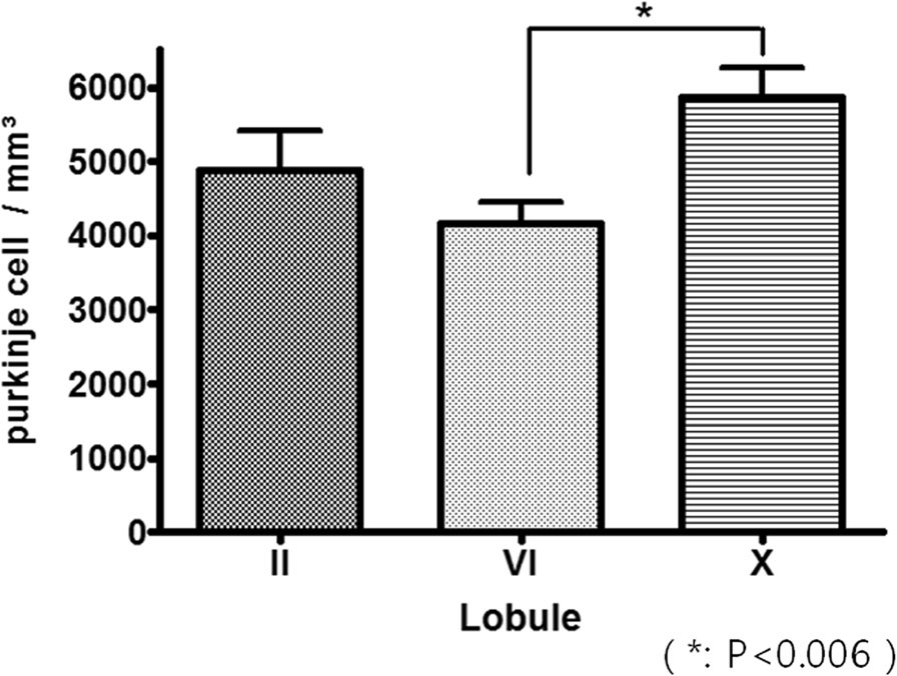
Purkinje cell density according to lobules. Counting frame size is 5.7 μm × 2.8 μm. (*: *p* < 0.006)

Two samples were randomly selected from the serial section of 150 μm thickness for each lobule of each experimental animal, and 40 serial sections of 1 μm thickness were obtained. The number density of each fifth section of the serial section was measured by physical disector (frame size; 300 μm x150μm). The number density of X lobule was the highest in palecerebellum (4888.89 ± 1157.75 pcs / mm^3^), neocerebellum (4158.73 ± 628.94 pcs / mm^3^) and (archicerebellum 5873.02 ± 854.79 pcs / mm^3^) (Fig. 4). Repeated-measures ANOVA revealed significant differences between the three lobules (*P* < 0.041), but there were no significant differences between the lobules in palecerebellum and neocerebellum and palecerebellum and archicerebellum (*P* < 0.30, *P* < 0.20). However, neocerebellum and archicerebellum showed significant differences (*P* < 0.006) (Fig. 4).

### Synaptic density in each lobule

The number density of synapses was 12.07 ± 1.38 synapse / 10 μm^3^ in II lobule, 12.39 ± 0.99 synapse / 10 μm^3^ in VI lobule, and 11.04 ± 1.85 synapse / 10 μm^3^ in X lobule. Repeated – measures ANOVA showed no significant difference between the three lobules (*P* < 0.435) (Fig. 3b).

### Synapse per of Purkinje cell in each lobule

**Fig. 5:**
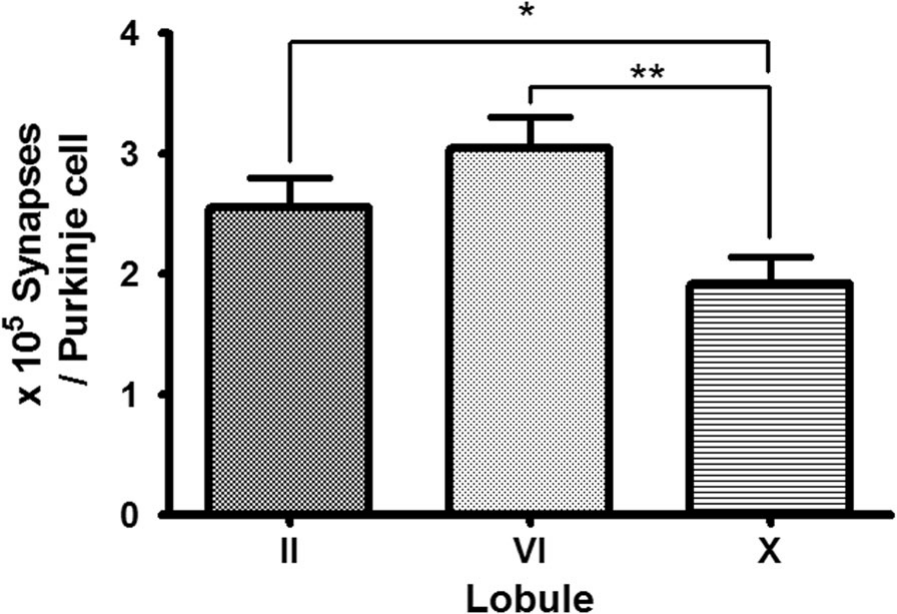
The number of synapses per Purkinje cell. (*: *p* < 0.06, **: *p* < 0.006)

By dividing the synaptic count density value of each lobule by the Purkinje cell count density (Nv (syn) / Nv (PC)), one cell can obtain the synaptic count density. The II lobule was 2.56 ± 0.53 × 10^5^ synapse / pc, the VIb lobule was 3.05 ± 0.60 × 10^5^ synapse / pc, and the X lobule was 1.92 ± 0.49 × 10^5^ synapse / pc (Fig. 5). Repeated – measures ANOVA showed significant differences among the three lobules (*P* < 0.018). There was no significant difference between lobule in palecerebellum and neocerebellum, and palecerebellum and archicerebellum (*P* < 0.30, *P* < 0.06). However, neocerebellum and archicerebellum showed significant differences (*P* < 0.006).

## Discussion

The cerebellum could be divided into archicerebellum, paleocerebellum and neocerebellum based on phylogeny. The archicerebellum is a vestibular cerebellum, paleocerebellum is a spinocerebellym, and neocerebellum is a pontocerebellum based on functional anatomical classifications. The vermis of the rodent cerebellum is divided into 10 lobules numbered I to X from rostral to caudal. Lobules of I to V are paleocerebellum, lobules of VI to IX are neocerebellum and lobule X is archicerebellum.

Purkinje cell, the largest cell in the cerebellum, has a diameter of about 20 ~ 30 μm. Many dendrites of Purkinje cells constitute cerebellar cortex and form synapses with parallel fibers from granule cells. There are many reports of the number of synapses that Purkinje cells are connected to parallel fibers (Napper and Harvey 1988a; Napper and Harvey 1988b). The density of synapse between Purkinje cell dendritic spines and parallel fiber varicosities were reported to be higher in neocerebellum than in archicerebellum (Heinsen and Heinsen 1983a) based on large number of electron microscopic data. But, the spine density of Purkinje cell based on high voltage electron microscopy did not show any difference among the phylogenic lobules (Park 2007).

In this study, the number of Purkinje cell in given volume was different among the lobules investigated. The Purkinje cell density in lobule X was higher compare to lobule VI. This is an interesting finding, which is agreement of the previous study by Heinsen and Heinsen (1983b) and the data studied human cerebellum (Skefos et al. 2014). Heinsen & Heinsen suggested that this Purkinje cell density difference according to the lobules is the evidence of evolutionary change of Purkinje cell density along the animal revolution in one animal brain (Heinsen and Heinsen 1983b). For example, the average density of Purkinje cell is 580~810/mm^2^ in rat (Heinsen and Heinsen 1983b), 510/mm^2^ in monkey (Fox and Barnard 1957), 300 mm^2^ in human (Braitenberg and Atwood 1958), which implies that density of Purkinje cell is negatively correlated with evolutionary course of the animal.

The Purkinje cell synapse density in this study revealed higher in lobule VI than lobule X, which corroborate the report of Heinsen and Heinsen (1983a). It is interesting that Purkinje cell density is lower in neocerebellum than archicerebellum, but synapse density between Purkinje cell dendritic spine and parallel fiber varicosity was higher in neocerebellum. This phenomenon could be understood as an analogy of the excavator and shovel; As a man driving excavator can outperform hundreds of people using shovel. Likewise, Purkinje cells in neocerebellum may outperform Purkinje cells in archicerebellum with their higher synaptic density. This discrepancy could be explained if morphometric data of the Purkinje cell dendritic trees and dendritic spine would be provided.

The difference in synaptic density implies that information is processed differently according to their phylogenic origins in addition to different electrophysiological characteristics (Kim et al. 2012; Witter and De Zeeuw 2015).

## Conclusion

In this study, the volume, number of Purkinje cells, and number of synapses in the lobule were calculated using the functional disector of the Stereo Investigator. The Purkinje cell density in archicerebellum was highest among the lobules studied, but the synaptic density between parallel fiber varicosity was higher in neocrebellum. This implies that morphological foot print of phylogeny exists in the cerebellar lobules.

## Data Availability

Not applicable. “Please contact author for data requests.”
